# Prevalence of hypertension among travelers and stability of blood pressure control during travel: a cross-sectional descriptive study and prospective cohort study

**DOI:** 10.1186/s40794-023-00199-5

**Published:** 2023-09-15

**Authors:** Watsapol Gultawatvichai, Wasin Matsee, Phimphan Pisutsan, Teera Kusolsuk, Udomsak Silachamroon, Chayasin Mansanguan, Saranath Lawpoolsri, Gerard T. Flaherty, Watcharapong Piyaphanee

**Affiliations:** 1 Department of Clinical Tropical Medicine, Faculty of Tropical Medicine, Mahidol University, 420/6 Ratchawithi Road, Bangkok, 10400 Thailand; 2 Department of Helminthology, Faculty of Tropical Medicine, Mahidol University, Bangkok, Thailand; 3 Department of Tropical Hygiene, Faculty of Tropical Medicine, Mahidol University, Bangkok, Thailand; 4 School of Medicine, University of Galway, Galway, Ireland; 5 National Institute for Prevention and Cardiovascular Health, Galway, Ireland

**Keywords:** Non-communicable diseases, Prevalence, Hypertension, Travelers, Blood pressure, Stability

## Abstract

**Background:**

Hypertension is a common and important risk factor for cardiovascular disease which is the leading cause of death among the general population and travelers. Data on hypertension among travelers are very limited due to the scarcity of research reports in this specific population. Therefore, this study aimed to determine the prevalence of hypertension among adult travelers and the stability of blood pressure control during international trips using a mobile automated blood pressure device.

**Methods:**

This was a cross-sectional descriptive study conducted at the Thai travel clinic, Hospital for Tropical Diseases in Bangkok, Thailand. All adult travelers completed a questionnaire which included demographic data, medical history, medication use, trip characteristics and hypertension awareness and knowledge. Standard two time blood pressure measurements were performed at the clinic to detect possible undiagnosed hypertension. Travelers with pre-existing hypertension were also invited to monitor their blood pressure level before and during their trip for a total of 14 days by using an automated blood pressure device and reporting the readings back to the study team.

**Result:**

During July and October 2022, a total of 1,359 adult travelers visited the Thai Travel Clinic before their international trip. The overall prevalence of hypertension was 28.8%, including those with pre-existing hypertension (6.7%) and those with newly diagnosed hypertension (22.2%). Travelers with newly diagnosed hypertension were significantly younger than travelers with pre-existing hypertension (38.5 years vs. 55.6 years, *p* < 0.001). Eleven travelers agreed to monitor their blood pressure, Most (90.9%, 10/11) had stable blood pressure control during their trip. One participant had > 10 mmHg higher blood pressure during the trip, however this was not clinically significant. All participants remained well, and acute symptoms secondary to hypertension were not reported.

**Conclusion:**

Up to 28.8% of adult travelers seen in pre-travel consultations had hypertension. Most of them were unaware of their blood pressure condition. Vital signs including blood pressure should be evaluated in all pre-travel visits in order to prevent undiagnosed severe hypertension that might lead to hypertensive crisis.

**Supplementary Information:**

The online version contains supplementary material available at 10.1186/s40794-023-00199-5.

## Background

Hypertension is a very common and significant medical problem worldwide. It is estimated that up to 26.4% of the global adult population had hypertension in 2000, and this prevalence is estimated to increase to 29.2% by 2025 [1, 2]. Uncontrolled hypertension is a major risk factor for cardiovascular disease and stroke which are among the leading causes of death among the general population worldwide. Cardiovascular disease is also the most common cause of death among international travelers [3–7].

Although international travel has been disrupted by the corona virus disease of 2019 pandemic, a recent report from the United Nations World Tourism Organization (UNWTO) showed that international travel has rebounded to 80% of pre-pandemic levels in the first quarter of 2023. It is estimated that international arrivals will recover to 95% of pre-pandemic levels by the end of this year [8, 9]. With more people traveling, it is likely that travelers with pre-existing medical conditions including hypertension will also embark on more international trips.

There is limited research reporting the prevalence of hypertension among travelers which varies between 5.2 to 18.6% depending on the population studied [10–13]. However, due to the lack of comprehensive data on this topic, it is difficult to determine the actual prevalence of hypertension among travelers. Moreover, little is known about the stability of blood pressure during travel. Theoretically, during international trips, travelers may experience various stresses including jet lag, lack of sleep, and flight delays [14]. These events might make their blood pressure more difficult to control. However, this is just a theoretical consideration; to date there has been no published evidence to support this assumption.

Therefore, we conducted this study aiming to determine the prevalence of hypertension among adult travelers and to determine their blood pressure variation during travel using an automated blood pressure device.

## Methods

### Study population and study site

This study was conducted in the Thai Travel Clinic, Hospital for Tropical Diseases, Bangkok, Thailand. Medical records of adult travelers (age ≥ 18 years) who visited the study site from July 2022 to October 2022 were reviewed. Records contained two parts, including a questionnaire section, which was completed by travelers before consultation, and a medical record section, which was filled by doctors and nurses at the clinic. Each record contained data on age, gender, nationality, destination, purpose of the trip, pre-existing medical conditions (if any), current medications (if any), vital signs (temperature, blood pressure, pulse rate).

All travelers were asked to rest before undergoing blood pressure measurement at the clinic. Travelers who had a history of hypertension as an underlying disease, regardless of the current blood pressure level and medical management status, were considered as having pre-existing hypertension. Travelers who had no history of hypertension but had two readings at the clinic with a systolic blood pressure (SBP) higher than 140 mmHg and/or diastolic blood pressure (DBP) higher than 90 mmHg after resting for 5 min were considered as having newly diagnosed hypertension (new hypertension).

All travelers who had hypertension (pre-existing and new) were invited to complete an additional questionnaire regarding hypertension. Information included lifestyle habit, smoking, alcohol consumption, physical activity, and salt intake was collected. Basic knowledge about hypertension, such as positive and negative factors for hypertension control and the possible complications of hypertension, was also included in the questionnaire (see Supplemental file).

### Variability of blood pressure during travel

Travelers with pre-existing hypertension were invited to monitor their blood pressure levels while traveling. Participants were requested to measure their blood pressure by using an automated blood pressure device two times a day—in the morning, before a meal, and at night, before going to bed.

An automated blood pressure device is used to measure and display arterial blood pressure through automated inflation and deflation of a cuff applied to an extremity. The method used to estimate blood pressure with an automated device is the detection of arterial flow through oscillometry. In this method, pulses sensed through the cuff are filtered, amplified, processed, and applied to an algorithm to estimate SBP and DBP. Participants could use any model of automated blood pressure device which was owned by them throughout their trip. Participants were advised that measurements should be taken while sitting on a chair with their feet on the floor, legs uncrossed, back supported, and with at least 5 min of relaxation before measurement. The fourteen-day data collection period was divided equally into before travel and during travel. Blood pressure levels were measured twice, with a 1–2-min interval between readings. Participants were required to measure their blood pressure levels at the same time every day during their travel, and were requested to submit their data to the investigator via email or using the official LINE® application, which is an online platform for communication with other individuals popular in Thailand.

The study protocol and questionnaire were reviewed and approved by the Ethics Committee of the Faculty of Tropical Medicine, Mahidol University.

### Sample size and statistical analysis

This study calculated the sample size for estimating the prevalence of hypertension among travelers, considering a prevalence rate of 13% and a desired precision of 5%. The expected sample size required for this estimate was determined to be at least 174 participants. For the secondary objective, the sample size was calculated to be at least 11, based on a desired precision of 5% and assuming a mean blood pressure difference of 10 mmHg and a standard deviation of 10 mmHg among travelers. The level of significance was set at 0.05 and the power at 0.80. A two-tailed Z-test was used with a Z-value of 1.96. Statistical analysis was performed using Statistical Products and Service Solutions (SPSS) software version 25.0. Categorical data were presented as numbers and percentages with a 95% confidence interval. Pearson's Chi-square test was used for comparing categorical data in the characteristics of hypertensive travelers, with a p-value below 0.05 considered statistically significant.

Instability of blood pressure during the trip was defined as a ≥ 10 mmHg change in mean SBP or mean DBP or mean arterial pressure (MAP) before and during travel or a SBP of ≥ 170 mmHg or DBP of ≥ 100 mmHg with a 20% change from the mean SBP or DBP.

## Results

Between July and October 2022, 1,359 adult travelers visited the Thai Travel Clinic before their international trip. Fifty-four percent of them were males, and the overall median age was 34 years (range 18–67 years). Their mean body mass index was 23.61 kg/m^2^ (SD ± 4.2). Up to 62.8% of them were Thai travelers, followed by North American/European (22.2%) and other non-Thai Asian travelers (11.8%). The main reason for travel was tourism (40.8%), followed by business/working (36.7%). Detailed demographic data are shown in Table 1.

**Table 1:** Characteristics of travelers at Thai travel clinic from July to October 2022

	**Total** (*n* = 1,359)	**Normal Blood Pressure**	**Total Hypertension**	**New Hypertension** **Prevalence (95%CI)**	**Pre-existing Hypertension** **Prevalence (95%CI)**
**Overall**	1359	967 (71.16%)	392 (28.84%)	301 (22.15%)	91 (6.70%)
Median age (range)	34 (18–91)	32 (18–91)	41.50 (18–87)	36 (18–79)	56 (31–87)
Mean age (SD)	37.07 (13.08)	34.88 (11.68)	42.47 (14.70)	38.49 (13.28)	55.65 (11.10)
Mean BMI (SD), *n* = 1,074	23.61 (4.22)	22.62 (3.62)	26.04(4.57)	25.89 (4.76)	26.54 (3.82)
Mean SBP(SD)	128.64 (16.98)	120.58 (11.02)	148.52(12.04)	149.44 (10.62)	145.45 (15.52)
Mean DBP(SD)	73.49 (12.58)	69.01 (9.19)	84.53(12.99)	84.84 (12.92)	83.48 (13.25)
**Age (years)**					
≤ 30	528	419 (79.36%)	109 (20.64%)	109 (20.64%)	0 (0%)
31–40	384	301 (78.39%)	83 (21.6%)	76 (19.79%)	7 (1.82%)
41–50	213	134 (62.91%)	79 (37.09%)	54 (25.35%)	25 (11.74%)
51–60	145	75 (51.72%)	70 (48.28%)	40 (27.59%)	30 (20.69%)
> 60	89	38 (42.70%)	51 (57.30%)	22 (24.72%)	29 (32.58%)
**BMI (kg/m**^**2**^**)**					
< 18.5	76	66 (86.84%)	10 (13.16%)	10 (3.88%)	0 (0%)
18.5–25	657	529 (80.52%)	128 (19.48%)	99 (15.07%)	29 (4.41%)
> 25	341	166 (48.68%)	175 (51.32%)	133 (39.00%)	42 (12.32%)
**Sex**					
Male	736	579 (78.67%)	157 (21.3%)	186 (25.27%)	49 (6.66%)
Female	623	388 (62.28%)	235 (37.72%)	115 (18.46%)	42 (6.74%)
**Nationality**					
Asian (Thai)	853	614 (71.98%)	239 (28.02%)	171 (20.05%)	68 (7.97%)
North American & European	302	210 (69.54%)	92 (30.46%)	79 (26.16%)	13 (4.30%)
Asian (Non-Thai)	160	112 (70.00%)	48 (30.0%)	39 (24.37%)	9 (5.63%)
Australian & Oceanic	33	22 (66.67%)	11 (33.3%)	10 (30.30%)	1 (3.03%)
South American & Others	11	9 (81.82%)	2 (18.2%)	2 (18.18%)	0 (0%)
**Purpose of travel**					
Tourism	555	383 (69.01%)	172 (30.99%)	134 (24.14%)	38 (6.85%)
Working	499	373 (74.75%)	126 (25.25%)	97 (19.44%)	29 (5.81%)
Studying	133	105 (78.95%)	28 (21.05%)	27 (20.30%)	1 (0.75%)
Visiting Friends and Relatives	132	88 (66.67%)	44 (33.33%)	33 (25.00%)	11 (8.33%)
Pilgrim	40	18 (45.00%)	22 (55.0%)	10 (25.00%)	12 (30.00%)
**Hypertension grade**					
Normal SBP < 129 and/or DBP < 84	732	717 (97.95%)	15 (2.05%)	0 (0%)	15 (2.05%)
High normal SBP 130–139 and/or DBP 85–89	267	250 (93.63%)	17 (6.37%)	0 (0%)	17 (6.37%)
Hypertension grade 1 SBP 140–159 and/or DBP 90–99	272	0 (0%)	272 (100.0%)	234 (86.03%)	38 (13.97%)
Hypertension grade 2 SBP 160–179 and/or DBP 100–109	71	0 (0%)	71 (100.0%)	55 (77.46%)	16 (22.54%)
Hypertension grade 3 SBP ≥ 180 and/or DBP ≥ 110	17	0 (0%)	17 (100.0%)	12 (70.59%)	5 (29.41%)

### Prevalence of hypertension among travelers in travel clinic

Among 1,359 travelers, 91 (6.7%) reported hypertension as their pre-existing disease, while 301 of 1359 travelers (22%) were considered to have newly diagnosed hypertension. The overall prevalence of hypertension was found to be 28.8%. The mean age of travelers with hypertension was significantly higher than travelers with normal blood pressure (34.9 years vs. 42.5 years, *p* < 0.005). The average body mass index (BMI) in travelers with hypertension was also significantly higher than the normal blood pressure group (26.0 vs 22.6 kg/m^2^, *p* < 0.05). Further details are shown in Table 1.

### Comparison between newly diagnosed hypertension and pre-existing hypertension

Travelers with newly diagnosed hypertension were significantly younger than travelers with pre-existing hypertension (38.5 years vs. 55.6 years, *p* < 0.001). The mean SBP in newly diagnosed hypertension was higher than travelers with pre-existing hypertension (149.4 mmHg vs. 145.5 mmHg, *p* < 0.01), while the mean DBP in both groups were not different.

Travelers with pre-existing hypertension reported dyslipidemia as a comorbidity more frequently when compared to travelers with newly diagnosed hypertension (37.3% vs. 5.3%, *p* < 0.001). This finding was also found in diabetes mellitus (DM), with 23.5% of travelers with pre-existing hypertension had DM while 2.6% of travelers with newly diagnosed hypertension had DM. However, no differences were observed between the two groups in term of salt intake, smoking, and physical exercise. Detailed traveler characteristics are shown in Table 2.

**Table 2:** Characteristics of hypertensive travelers at Thai travel clinic from July to October 2022

	**Total** ***N*= 89 (%)**	**New hypertension** ***N* = 38 (%)**	**Pre-existing hypertension** ***N* = 51 (%)**
**Age (YEARS)**			*P* < 0.001
** < 30**	11 (12.4)	11 (28.9)	0 (0)
**31–40**	16 (18.0)	10 (26.3)	6 (11.8)
**41–50**	20 (22.5)	7 (18.4)	13 (25.5)
**51–60**	24 (27.0)	7 (18.4)	17 (33.3)
** > 60**	18 (20.2)	3 (7.9)	15 (29.4)
**BMI (KG/M**^**2**^**)**			*P* = 0.550
** < 18.5**	1 (1.1)	1 (2.6)	0 (0)
**18.5–25**	29 (32.6)	12 (31.6)	17 (33.3)
** > 25**	58 (65.3)	25 (65.8)	33 (64.7)
**Sex**			*P* = 0.517
Male	48 (53.9)	22 (57.9)	26 (51.0)
Female	41 (46.1)	16 (42.1)	25 (49)
**Nationality**			*P* = 0.564
Thai	66 (74.2)	27 (71.1)	39 (76.5)
Non-Thai	23 (25.8)	11 (28.9)	12 (23.5)
**Smoking**			*P* = 0.032
No smoking	73 (82.0)	30 (78.3)	43 (84.3)
Former smoker	7 (7.9)	1 (2.6)	6 (11.8)
Smoking	9 (10.1)	7 (18.4)	2 (3.9)
**Physical exercise**			*P* = 0.279
**0** min/week	28 (31.5)	15 (39.5)	13 (25.5)
** < 90** min**/**week	26 (29.2)	8 (21.1)	18 (35.3)
**90–150** min**/**week	20 (22.5)	10 (26.3)	10 (19.6)
** > 150** min**/**week	15 (16.9)	5 (13.2)	10 (19.6)
**Salt diet**			*P* = 0.681
Sodium diet ≤ 2.4 g/day	40 (44.9)	17 (44.7)	23 (45.1)
Sodium diet > 2.4 g/day	48 (53.9)	21 (55.3)	27 (52.9)
Missing	1 (1.1)	0 (0)	1 (2.00)
**Sleep hours**			*P* = 0.642
< 5 h	11 (12.4)	4 (10.5)	7 (13.7)
5–9 h	75 (84.3)	32 (84.2)	43 (84.3)
> 9 h	3 (3.4)	2 (5.3)	1 (2)
**Alcohol**			*P* = 0.158
No alcohol drinking	44 (49.4)	16 (42.1)	28 (54.9)
Former	8 (9.0)	2 (5.3)	6 (11.8)
Alcohol drinking	37 (41.6)	20 (52.6)	17 (33.3)
**Family history of CVD**			*P* = 0.003
Yes	32 (36.0)	7 (18.4)	25 (49.0)
No	57 (64)	31 (81.6)	26 (51.0)
**Comorbidity**			
**DLP**			*P* < 0.001
Yes	21 (23.6)	2 (5.3)	19 (37.3)
No	68 (76.4)	36 (94.7)	32 (62.7)
**MI**			
No	89 (100)	38 (100)	51 (100)
**Stroke**			
No	89 (100)	38(100)	51 (100)
**DM**			*P* = 0.006
Yes	13 (14.6)	1 (2.6)	12 (23.5)
No	76 (85.4)	37 (97.4)	39 (76.5)
**CKD**			*P* = 1.000
Yes	1(1.12)	0 (0)	1 (2)
No	88(98.88)	38 (100)	50 (98)
**Knowledge score**			*P* = 0.360
0–2 points (lack of)	9(10.1)	4(10.5)	5(9.8)
3–5 points (moderate)	42(47.2)	21(55.3)	21(41.2)
6–8 points (good)	38(42.7)	13(34.2)	25(49.0)

*Abbreviations*: *CVD*  Cardiovascular diseases, *MI* Myocardial infarction, *DM* Diabetes mellitus, *DLP* Dyslipidemia

### Measurement and variability of blood pressure pre- and during the trip

Although twenty-two travelers with pre-existing hypertension consented and agreed to measure their blood pressure before and during the trip, half of them (11/22) adhered to the research protocol and reported their blood pressure data to the study team.

**Fig. 1:**
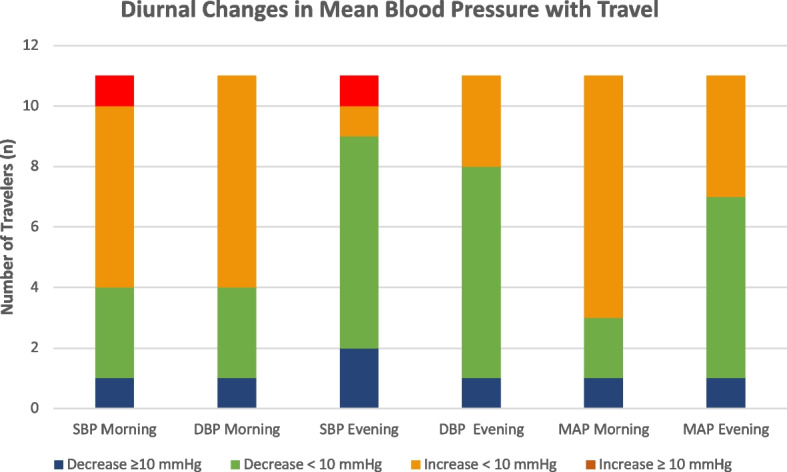
Diurnal changes in mean blood pressure readings in hypertensive travelers with travel

Among 11 participants, all of them were Thai, with an overall mean age of 56.9 years. Five of them were male (45.5%), while six of them were female (54.5%). Nearly all participants (10/11, 90.9%) had at least one medication for hypertension. Their mean SBP, DBP and MAP in the morning before the trip were similar to their mean SBP, DBP, MAP during the trip. When considering individual blood pressure levels, there was only one traveler (participant no 9) with a high SBP in the morning and evening which matched our criteria for instability of blood pressure (mean SBP change ≥ 10 mmHg) during the trip. However, no subject had a very high blood pressure episode (SBP ≥ 160 mmHg or DBP ≥ 100 mmHg) during the trip. No acute events related to hypertension (syncope, hypertensive crisis, MI, stroke) were reported from the travelers during this trip. Further details about the variability of blood pressure values of all participants are shown in Tables 3 and 4 and in Fig. 1.

**Table 3:** Mean morning blood pressure values before and during travel

	**Mean SBP Morning (mmHg)**		**Mean DBP Morning** **(mmHg)**		**Mean MAP Morning** **(mmHg)**	
Participant	Before Travel	During Travel	Before Travel	During Travel	Before Travel	During Travel
1	139.83	142.14	96.33	101.71	110.83	115.19
2	144.93	146.29	95.71	98.86	112.12	114.67
3	132.00	129.07	83.50	83.57	99.67	98.74
4	113.67	107.14	75.50	67.14	88.22	80.48
5	122.00	125.07	73.57	77.21	89.71	93.17
6	133.29	135.67	78.64	81.58	96.86	99.61
7	132.67	140.75	85.50	82.50	101.22	101.92
8	152.17	134.57	101.17	82.00	118.17	99.52
9	120.07	131.30	62.93	67.60	81.98	88.83
10	128.29	128.00	83.00	84.83	98.10	99.22
11	133.43	140.14	86.43	84.21	102.10	102.86
**MEAN (SD)**	132.03 (11.10)	132.74 (10.75)	83.84 (11.17)	82.84 (10.64)	99.91 (10.88)	99.47 (10.05)

**Table 4:** Mean evening blood pressure values before and during travel

	**Mean SBP Evening (mmHg)**		**Mean DBP Evening** **(mmHg)**		**Mean MAP Evening** **(mmHg)**	
**Participant**	**Before** **Travel**	**During** **Travel**	**Before** **Travel**	**During** **Travel**	**Before** **Travel**	**During** **Travel**
1	140.67	132.43	94.50	88.14	109.89	102.90
2	137.86	131.50	91.43	85.57	105.55	100.88
3	141.00	128.83	85.50	81.67	104.00	97.39
4	112.38	101.64	70.75	62.50	84.63	75.55
5	122.21	121.43	74.00	73.29	88.81	89.33
6	119.50	119.07	71.14	75.14	86.07	89.79
7	128.75	126.00	76.00	72.14	93.58	90.10
8	141.58	134.71	95.58	80.00	110.92	98.24
9	117.57	129.08	58.57	61.42	76.83	83.97
10	128.86	125.00	85.00	84.33	99.62	97.89
11	134.57	139.86	80.36	83.71	96.98	102.43
**Mean (SD)**	129.54 (10.43)	126.32 (10.10)	80.26 (11.46)	77.08 (9.06)	96.08 (11.12)	93.50 (8.57)

## Discussion

To our knowledge, this is the first study focused on the prevalence of hypertension among travelers in Thailand. In our study, the overall prevalence of hypertension was 28.84%. Interestingly, this prevalence was much higher than other reports. A previous study from a travel clinic in Marseille, France, reported a prevalence of 4.6%, while a study from Galway, Ireland revealed a self-reported prevalence of 2.9% [10, 15]. The high prevalence of hypertension in our study may be a result of including both individuals with pre-existing hypertension, and newly diagnosed hypertension. while most previous studies included travelers with pre-existing hypertension only [10, 15].

It is reasonable to suggest that the prevalence of newly diagnosed hypertension in our study was overestimated due to the “White Coat Hypertension” phenomenon. This phenomenon is characterized by an elevated blood pressure reading in a medical facility, while the individual’s ambulatory blood pressure remains within the normal range [16]. Moreover, although we complied with standard blood pressure measurement guidelines (two measurements with adequate rest) and standard criteria to diagnose hypertension, we used a single clinic visit to diagnose hypertension. Several studies suggest that single clinic visits tended to overestimate the prevalence of hypertension [17–20]. A large study in China showed that the prevalence of hypertension at one visit was 18.1%, however this dropped to 9.47% following a second visit [19]. Another study from Switzerland showed a similar result which reported that the prevalence of hypertension between one and two visits decreased by 13% (from 33.8 to 29.5) [20].

Although the prevalence of newly diagnosed hypertension in our study (22.15%) was likely to be overestimated, we could not totally ignore this prevalence. If we assume the effect of a single clinic visit and white coat hypertension was around 50% overestimated, at a conservative estimate, we can still observe an 11% prevalence of newly diagnosed hypertension in our group (mean age 38.5 years). This value is comparable to the prevalence in the general population in the same age group. Recent reports from the National Center for Health Statistics, United States showed that people aged 18–39 years had a 22.4% prevalence of hypertension [21], while a survey in Thailand reported a 17% prevalence of hypertension in the 30–44 year age group [22]. These findings indicate that travelers should undergo screening for undiagnosed hypertension.

It is also important to note that, according to the World Health Organization, up to 46% of adults with hypertension are unaware that they have the condition, as healthy individuals may not have their blood pressure measured for many years [23]. Travel medicine practitioners have an opportunity to detect this common medical problem when they see travelers during a pre-travel consultation. Vital signs including blood pressure should be evaluated in all travel clinic visits as a basic standard. The findings from this study suggest that DLP and DM should be evaluated in travelers with pre-existing hypertension.

The prevalence of travelers with pre-existing hypertension in our study was 6.7%. Our prevalence was higher when compared to that reported from France (4.6%) and Ireland (2.9%) [10, 15]. One possible explanation for this discrepancy could be the age difference between the populations studied. It is well known that the prevalence of hypertension increases with age. In our study, the average age among travelers was 37.1 years, while the average ages among travelers in the French and Irish studies were 31.7 and 36.6 years, respectively. Other reports also show a similar trend, such as another French study which reported a prevalence of hypertension of 25.3% among Hajj pilgrims (average age 58 years) [24].

### Blood pressure control before and during travel

Among the 11 participants that agreed to participate in the blood pressure monitoring arm of the study, we found that most of them had well controlled blood pressure before and during the trip. There was no significant change in SBP, DBP, MAP throughout the trip. Although their blood pressure in the morning was slightly higher than in the evening, this was not clinically relevant (less than 10 mmHg). This finding may be due to the phenomenon known as the “morning surge of blood pressure”. This has been documented in various reviews as a normal aspect of human physiology due to the effect of circadian rhythm, which can cause high blood pressure levels in the morning [25, 26]. Another factor that might have led to higher blood pressure in the morning was the medications taken, since most travelers measure their blood pressure before taking their antihypertensive medication. It is well known that anti-hypertensive medication might take several hours to reach its maximum effect, and this might be the reason why blood pressure readings seem to be more favorable in the morning. However, with modern antihypertensive medication with longer half lives (once daily dosing), the effect of timing would be much less significant. Another aspect to consider that may have impacted data accuracy is the use of different mobile automated blood pressure devices for each participant, which made it difficult to calibrate the devices and maintain consistency in measurements.

Although it is reasonable to assume that during international travel, travelers might have difficulty controlling their blood pressure due to several reasons, we could not demonstrate that in our study. Over 90% (10/11) travelers had stable blood pressure control during the trip, while only one participant had clinically insignificant higher blood pressure (≥ 10 mmHg). Moreover, all participants remained well, and no hypertension-related clinical events were reported during the trip.

Nevertheless, it is important to keep in mind that international travel can be stressful due to a variety of reasons including airport stress, lack of sleep, jet lag, and language/communication barriers, all of which can negatively impact blood pressure control. Dietary habits may also adversely impact blood pressure regulations, especially when consuming foods and beverages high in salt, caffeine, and alcohol. Those factors can contribute to an increase in blood pressure levels [14, 27, 28].

Travelers with pre-existing hypertension should have their blood pressure under control before the trip and continue taking their own antihypertensive drug(s) throughout the trip. Failure to take medication is always the main reason that makes blood pressure uncontrollable. Severely high BP or marked variation of blood pressure during the trip can lead to serious or even fatal consequences.

### Limitations of the study

There are several limitations to this study. Firstly, newly diagnosed cases of hypertension were determined based on high blood pressure readings taken at two separate timepoints within a single visit, which may lead to overestimation when compared to using multiple measurements over several visits or using ambulatory blood pressure monitoring. Secondly, participants enrolled in the second arm of the study were a unique group with well-controlled blood pressure before the trip, very good compliance with their doctor’s advice, and use of their own blood pressure measurement device. The results relating to blood pressure stability during the trip in this group might not, therefore, be generalisable to other hypertensive travelers.

## Conclusions

Our study found that hypertension was common among adult travelers, with an overall prevalence of 28.8%. Most travelers with hypertension were unaware of their high blood pressure level before visiting our travel clinic. Travelers with pre-existing hypertension in our study had relatively stable blood pressure throughout the trip. However, given that hypertension is an important risk factor for serious cardiovascular events or stroke, travel medicine practitioners should always assess travelers’ blood pressure in a pre-travel health consultation. In the next phase of our study, focusing on monitoring blood pressure, we aim to enhance the reliability of our findings by increasing the participant sample size. We will aim to use the same model of automated blood pressure device throughout the study to ensure consistency and accuracy. These steps will help us obtain more applicable results regarding blood pressure monitoring among travelers.

### Supplementary Information

**Additional file 1.** 

## Data Availability

The datasets used and/or analysed during the current study are available from the corresponding author on reasonable request.

## Abbreviations

BMI: Body Mass Index
CI: Confidence Interval
CKD: Chronic Kidney Disease
DBP: Diastolic Blood Pressure
DLP: Dyslipidemia
DM: Diabetes Mellitus
MAP: Mean Arterial Blood Pressure
MI: Myocardial Infarction
SBP: Systolic Blood Pressure
SD: Standard Deviation
SPSS: Statistical Products and Service Solutions
UNWTO: United Nations World Tourism Organization
