# Supplementary material for: Prevalence of hypertension among travelers and stability of blood pressure control during travel: a cross-sectional descriptive study and prospective cohort study
Source: Trop Dis Travel Med Vaccines. 2023 Sep 15;9:13. doi: 10.1186/s40794-023-00199-5 (PMC10503084; doi:10.1186/s40794-023-00199-5)
Supplement: Supplementary file 1 — Additional file 1. [file 40794_2023_199_MOESM1_ESM.pdf]

Patient ID | | | |

THE PREVALENCE OF HYPERTENSION AMONG TRAVELERS AND STABILITY OF BLOOD PRESSURE CONTROL DURING TRIP

APPROVED
ETHICS COMMITTEE
FACULTY OF TROPICAL MEDICINE
MAHIDOL UNIVERSITY
Digitally signed by Faculty of Tropical Medicine, Mahidol University
DN: c=TH, st=Bangkok, l=Ratchathewi, o=Faculty of Tropical Medicine, Mahidol University, cn=Faculty of Tropical Medicine, Mahidol University, email=tmectropmed@mahidol.ac.th
Date: 2022.07.04 11:49:24 +07'00'

Part 1. 1 Demographic data

Baseline characteristics	
Visit dated (DD-MMM-YYYY)	[__|__] [__|__|__] [__|__|__|__]
1. Age (years)	[__|__]
2. Gender:	☐$_1$ Male ☐$_2$ Female
3. Nationality:	☐$_1$ Thai ☐$_2$ non-Thai ,Please specify____________
4. Blood pressure at Thai Travel clinic	[__|__|__] /[__|__|__] mmHg (initial) [__|__|__] /[__|__|__] mmHg (repeated after 5 mins)
5. Weight	[__|__|__].[__]Kg.
6. Height	[__|__|__] cm.
5. Smoking	☐$_1$ no ☐$_2$ former ☐$_3$current____________pack/day
6. Alcohol drinking<br>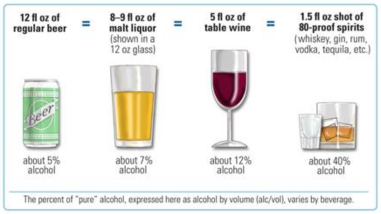 	☐$_1$ no ☐$_2$ former ☐$_3$current____________drink/day
7. Physical exercise and physical activity<br>(Moderate to vigorous exercise)	☐$_1$ no ☐$_2$ <90mins/week<br>☐$_3$ 90-150mins/week ☐$_4$ >150mins/week
8. Underlying disease	☐$_1$ no ☐$_2$ Yes, please answer 8.1-8.5
8.1 Hypertension	☐$_1$ no ☐$_2$ yes, please answer 8.1.1
8.1.1 on Hypertension drug(s)	☐$_1$ no ☐$_2$ yes , please specify__________________________<br>____

Patient ID | | | | |

**Baseline characteristics**	
8.1.2 Do you take hypertension drug(s) regularly?	$\square_1$ no $\square_2$ yes
8.2 Diabetes mellitus	$\square_1$ no $\square_2$ yes
8.3 Chronic kidney disease	$\square_1$ no $\square_2$ yes
8.4 Stroke	$\square_1$ no $\square_2$ yes
8.5 Myocardial infarction	$\square_1$ no $\square_2$ yes
8.6 Other diseases	$\square_1$ no $\square_2$ yes ______________________________
9. Sleeping hours per day	$\square_1$ <5 hrs. $\square_2$ 5-9 hrs. $\square_2$ >9 hrs.
10. Salt diet (>6 g/day or 2.4g sodium or 1 teaspoons)<br>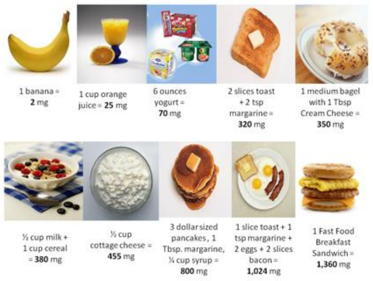 	$\square_1$ no $\square_2$ yes
11. purpose of this trip	$\square_1$ leisure $\square_2$ business/working $\square_3$ visit family/go back home<br>$\square_4$ others________________
12. Do you have any family history of cardiovascular diseases	$\square_1$ no $\square_2$ yes

APPROVED
ETHICS COMMITTEE
FACULTY OF TROPICAL MEDICINE
MAHIDOL UNIVERSITY

Patient ID | | | |

THE PREVALENCE OF HYPERTENSION AMONG TRAVELERS AND STABILIT

Y OF BLOOD PRESSURE CONTROL DURING TRIP

Part 1. 2 Hypertension Awareness and knowledge questionnaire

1. Is this your first time that your blood pressure is >140/90mmHg?	☐$_1$ no ☐$_2$ yes
2. Losing weight usually makes blood pressure	☐$_1$ decrease ☐$_2$ increase ☐$_3$ same ☐$_4$ don't know
3. Eating less salt usually make blood pressure	☐$_1$ decrease ☐$_2$ increase ☐$_3$ same ☐$_4$ don't know
4. High blood pressure can cause of heart attack	☐$_1$ no ☐$_2$ yes ☐$_3$ don't know
5. High blood pressure can cause of stroke	☐$_1$ no ☐$_2$ yes ☐$_3$ don't know
6. High blood pressure can cause of kidney problems	☐$_1$ no ☐$_2$ yes ☐$_3$ don't know
7. High blood pressure can cause of diabetes	☐$_1$ no ☐$_2$ yes ☐$_3$ don't know
8. Smoking a pack of cigarettes per day is a risk factor for hypertension	☐$_1$ no ☐$_2$ yes ☐$_3$ don't know
9. Moderate exercise 30mins/day 3-5 times a week is a risk factor for hypertension	☐$_1$ no ☐$_2$ yes ☐$_3$ don't know

APPROVED
ETHICS COMMITTEE
FACULTY OF TROPICAL MEDICINE
MAHIDOL UNIVERSITY

Patient ID | | | |

Part 2. 1 Blood Pressure monitoring before trip

Current Medication____

____

____

____

Date	Morning		Evening	
	1st	2nd	1st	2nd
Day 1	Blood pressure<br>_______/________<br>Pulse____________	Blood pressure<br>_______/________<br>Pulse____________	Blood pressure<br>_______/________<br>Pulse____________	Blood pressure<br>_______/________<br>Pulse____________
	Time_______:______<br>Country___________<br>City_____________<br>Activity__________	Time_______:______<br>Country___________<br>City_____________<br>Activity__________	Time_______:______<br>Country___________<br>City_____________<br>Activity__________	Time_______:______<br>Country___________<br>City_____________<br>Activity__________
Day 2	Blood pressure<br>_______/________<br>Pulse____________	Blood pressure<br>_______/________<br>Pulse____________	Blood pressure<br>_______/________<br>Pulse____________	Blood pressure<br>_______/________<br>Pulse____________
	Time_______:______<br>Country___________<br>City_____________<br>Activity__________	Time_______:______<br>Country___________<br>City_____________<br>Activity__________	Time_______:______<br>Country___________<br>City_____________<br>Activity__________	Time_______:______<br>Country___________<br>City_____________<br>Activity__________
Day 3	Blood pressure<br>_______/________<br>Pulse____________	Blood pressure<br>_______/________<br>Pulse____________	Blood pressure<br>_______/________<br>Pulse____________	Blood pressure<br>_______/________<br>Pulse____________
	Time_______:______<br>Country___________	Time_______:______<br>Country___________	Time_______:______<br>Country___________	Time_______:______<br>Country___________

APPROVED
ETHICS COMMITTEE
FACULTY OF TROPICAL MEDICINE
MAHIDOL UNIVERSITY

Patient ID | | | |

	City______________<br>Activity___________	City______________<br>Activity___________	City______________<br>Activity___________	City______________<br>Activity___________
Day 4	Blood pressure<br>________/_________<br>Pulse_____________	Blood pressure<br>________/_________<br>Pulse_____________	Blood pressure<br>________/_________<br>Pulse_____________	Blood pressure<br>________/_________<br>Pulse_____________
	Time_______:______<br>Country___________<br>City______________<br>Activity___________	Time_______:______<br>Country___________<br>City______________<br>Activity___________	Time_______:______<br>Country___________<br>City______________<br>Activity___________	Time_______:______<br>Country___________<br>City______________<br>Activity___________
Day 5	Blood pressure<br>________/_________<br>Pulse_____________	Blood pressure<br>________/_________<br>Pulse_____________	Blood pressure<br>________/_________<br>Pulse_____________	Blood pressure<br>________/_________<br>Pulse_____________
	Time_______:______<br>Country___________<br>City______________<br>Activity___________	Time_______:______<br>Country___________<br>City______________<br>Activity___________	Time_______:______<br>Country___________<br>City______________<br>Activity___________	Time_______:______<br>Country___________<br>City______________<br>Activity___________
Day 6	Blood pressure<br>________/_________<br>Pulse_____________	Blood pressure<br>________/_________<br>Pulse_____________	Blood pressure<br>________/_________<br>Pulse_____________	Blood pressure<br>________/_________<br>Pulse_____________
	Time_______:______<br>Country___________<br>City______________<br>Activity___________	Time_______:______<br>Country___________<br>City______________<br>Activity___________	Time_______:______<br>Country___________<br>City______________<br>Activity___________	Time_______:______<br>Country___________<br>City______________<br>Activity___________
Day 7	Blood pressure<br>________/_________<br>Pulse_____________	Blood pressure<br>________/_________<br>Pulse_____________	Blood pressure<br>________/_________<br>Pulse_____________	Blood pressure<br>________/_________<br>Pulse_____________
	Time_______:______<br>Country___________<br>City______________<br>Activity___________	Time_______:______<br>Country___________<br>City______________<br>Activity___________	Time_______:______<br>Country___________<br>City______________<br>Activity___________	Time_______:______<br>Country___________<br>City______________<br>Activity___________

APPROVED
ETHICS COMMITTEE
FACULTY OF TROPICAL MEDICINE
MAHIDOL UNIVERSITY

Patient ID | | | |

Part 2. 2 Blood Pressure monitoring during trip

Current Medication____

____

____

____

Date	Morning		Evening	
	1st	2nd	1st	2nd
Day 1	Blood pressure<br>________/_________<br>Pulse______________	Blood pressure<br>________/_________<br>Pulse______________	Blood pressure<br>________/_________<br>Pulse______________	Blood pressure<br>________/_________<br>Pulse______________
	Time_______:______<br>Country___________<br>City_______________<br>Activity___________	Time_______:______<br>Country___________<br>City_______________<br>Activity___________	Time_______:______<br>Country___________<br>City_______________<br>Activity___________	Time_______:______<br>Country___________<br>City_______________<br>Activity___________
Day 2	Blood pressure<br>________/_________<br>Pulse______________	Blood pressure<br>________/_________<br>Pulse______________	Blood pressure<br>________/_________<br>Pulse______________	Blood pressure<br>________/_________<br>Pulse______________
	Time_______:______<br>Country___________<br>City_______________<br>Activity___________	Time_______:______<br>Country___________<br>City_______________<br>Activity___________	Time_______:______<br>Country___________<br>City_______________<br>Activity___________	Time_______:______<br>Country___________<br>City_______________<br>Activity___________
Day 3	Blood pressure<br>________/_________<br>Pulse______________	Blood pressure<br>________/_________<br>Pulse______________	Blood pressure<br>________/_________<br>Pulse______________	Blood pressure<br>________/_________<br>Pulse______________
	Time_______:______<br>Country___________	Time_______:______<br>Country___________	Time_______:______<br>Country___________	Time_______:______<br>Country___________

APPROVED
ETHICS COMMITTEE
FACULTY OF TROPICAL MEDICINE
MAHIDOL UNIVERSITY

Patient ID | | | |

	City_______________<br>Activity____________	City_______________<br>Activity____________	City_______________<br>Activity____________	City_______________<br>Activity____________
Day 4	Blood pressure<br>_________/__________<br>Pulse______________	Blood pressure<br>_________/__________<br>Pulse______________	Blood pressure<br>_________/__________<br>Pulse______________	Blood pressure<br>_________/__________<br>Pulse______________
	Time________:______<br>Country____________<br>City_______________<br>Activity____________	Time________:______<br>Country____________<br>City_______________<br>Activity____________	Time________:______<br>Country____________<br>City_______________<br>Activity____________	Time________:______<br>Country____________<br>City_______________<br>Activity____________
Day 5	Blood pressure<br>_________/__________<br>Pulse______________	Blood pressure<br>_________/__________<br>Pulse______________	Blood pressure<br>_________/__________<br>Pulse______________	Blood pressure<br>_________/__________<br>Pulse______________
	Time________:______<br>Country____________<br>City_______________<br>Activity____________	Time________:______<br>Country____________<br>City_______________<br>Activity____________	Time________:______<br>Country____________<br>City_______________<br>Activity____________	Time________:______<br>Country____________<br>City_______________<br>Activity____________
Day 6	Blood pressure<br>_________/__________<br>Pulse______________	Blood pressure<br>_________/__________<br>Pulse______________	Blood pressure<br>_________/__________<br>Pulse______________	Blood pressure<br>_________/__________<br>Pulse______________
	Time________:______<br>Country____________<br>City_______________<br>Activity____________	Time________:______<br>Country____________<br>City_______________<br>Activity____________	Time________:______<br>Country____________<br>City_______________<br>Activity____________	Time________:______<br>Country____________<br>City_______________<br>Activity____________
Day 7	Blood pressure<br>_________/__________<br>Pulse______________	Blood pressure<br>_________/__________<br>Pulse______________	Blood pressure<br>_________/__________<br>Pulse______________	Blood pressure<br>_________/__________<br>Pulse______________
	Time________:______<br>Country____________<br>City_______________<br>Activity____________	Time________:______<br>Country____________<br>City_______________<br>Activity____________	Time________:______<br>Country____________<br>City_______________<br>Activity____________	Time________:______<br>Country____________<br>City_______________<br>Activity____________

APPROVED
ETHICS COMMITTEE
FACULTY OF TROPICAL MEDICINE
MAHIDOL UNIVERSITY
